# Anti-Inflammatory Effect of Neoechinulin A from the Marine Fungus *Eurotium* sp. SF-5989 through the Suppression of NF-кB and p38 MAPK Pathways in Lipopolysaccharide-Stimulated RAW264.7 Macrophages

**DOI:** 10.3390/molecules181113245

**Published:** 2013-10-25

**Authors:** Kyoung-Su Kim, Xiang Cui, Dong-Sung Lee, Jae Hak Sohn, Joung Han Yim, Youn-Chul Kim, Hyuncheol Oh

**Affiliations:** 1 College of Pharmacy, Wonkwang University, Iksan 570-749, Korea; E-Mails: pipo5@wku.ac.kr (K.-S.K); loodia@wku.ac.kr (X.C.); hsds@wku.ac.kr (D.-S.L.); 2 Standardized Material Bank for New Botanical Drugs, College of Pharmacy, Wonkwang University, Iksan 570-749, Korea; 3 Key Laboratory of Natural Resources and Functional Molecules of the Changbai Mountain, Affiliated Ministry of Education, Yanbian University College of Pharmacy, 977 Gongyuan Road, Yanji 133002, Jilin, China; 4 Hanbang Body-Fluid Research Center, Wonkwang University, Iksan 570-749, Korea; 5 College of Medical and Life Sciences, Silla University, Busan 617-736, Korea; E-Mail: jhsohn@silla.ac.kr (J.H.S.); 6 Korea Polar Research Institute, KORDI, 7-50 Songdo-dong, Yeonsu-gu, Incheon 406-840, Korea; E-Mail: jhyim@kopri.re.kr (J.H.Y.)

**Keywords:** neoechinulin A, *Eurotium rubrum*, RAW264.7 macrophages, inflammation, NF-κB, MAPK

## Abstract

In the course of a bioassay-guided study of metabolites from the marine fungus *Eurotium* sp. SF-5989, two diketopiperazine type indole alkaloids, neoechinulins A and B, were isolated. In this study, we investigated the anti-inflammatory effects of neoechinulins A (**1**) and B (**2**) on lipopolysaccharide (LPS)-stimulated RAW264.7 macrophages. Neoechinulin A (**1**) markedly suppressed the production of nitric oxide (NO) and prostaglandin E_2 _(PGE_2_) and the expression of inducible nitric oxide synthase (iNOS) and cyclooxygenase-2 (COX-2) in a dose dependent manner ranging from 12.5 µM to 100 µM without affecting the cell viability. On the other hand, neoechinulin B (**2**) affected the cell viability at 25 µM although the compound displayed similar inhibitory effect of NO production to neoechinulin A (**1**) at lower doses. Furthermore, neoechinulin A (**1**) decreased the secretion of pro-inflammatory cytokines, such as tumor necrosis factor-α (TNF-α) and interleukin-1β (IL-1β). We also confirmed that neoechinulin A (**1**) blocked the activation of nuclear factor-kappaB (NF-κB) in LPS-stimulated RAW264.7 macrophages by inhibiting the phosphorylation and degradation of inhibitor kappa B (IκB)-α. Moreover, neoechinulin A (**1**) decreased p38 mitogen-activated protein kinase (MAPK) phosphorylation. Therefore, these data showed that the anti-inflammatory effects of neoechinulin A (**1**) in LPS-stimulated RAW264.7 macrophages were due to the inhibition of the NF-κB and p38 MAPK pathways, suggesting that neoechinulin A (**1**) might be a potential therapeutic agent for the treatment of various inflammatory diseases.

## 1. Introduction

Inflammation is a defense mechanism of the body in response to various stimuli such as irradiation, physical damage and metabolic overload [1]. However, prolonged or deregulated inflammatory responses can lead to a variety of diseases including arthritis, hepatitis, septic shock syndrome, and neurodegenerative disorders [2,3]. Macrophages play a crucial role in several inflammatory responses [4]. Lipopolysaccharide (LPS) activates macrophages to produce pro-inflammatory mediators and pro-inflammatory cytokines, including nitric oxide (NO), prostaglandin E_2_ (PGE_2_), tumor necrosis factor-α (TNF-α), and interleukin-1β (IL-1β) [5,6]. Inducible nitric oxide synthase (iNOS) catalyzes the formation of excessive nitric oxide (NO), which can lead to the development of inflammatory diseases [7]. Cyclooxygenase-2 (COX-2), an inducible enzyme, is induced by pro-inflammatory stimuli and responsible for the synthesis of PGE_2 _ [8]. Therefore, the overproduction of NO and PGE_2_ by iNOS and COX-2 plays a critical role in the regulation of the inflammatory process. 

The transcription factor nuclear factor-kappa B (NF-κB) is an important regulator of inflammatory responses [9]. In cells under normal conditions, NF-κB exists in an inactive form in a complex with its inhibitory protein (IκB) in the cytoplasm. Upon activation by various stimuli, including TNF-α and LPS, the IκB protein is phosphorylated and degraded resulting in free NF-κB, which translocates to the nucleus [10]. Once in the nucleus, NF-κB binds to DNA binding sites to regulate the transcription of its target genes, leading to the transcription of pro-inflammatory mediators and cytokines including iNOS, COX-2, NO, PGE_2_, TNF-α, and IL-1β [11,12].

The mitogen-activated protein kinases (MAPKs) are one of the major kinase families associated with cellular processes such as differentiation, stress responses, apoptosis, and immune defense [13]. There are three major subgroups of MAPKs, including extracellular signal-regulated kinase (ERK), c-Jun N-terminal kinase (JNK), and p38 MAPK. They play a crucial role in inducing cytokine production [14] and the expression of iNOS and COX-2 [15]. Therefore, NF-κB and MAPK are critical factors in the inflammatory process and important targets for anti-inflammatory molecules.

Marine-derived fungi have proven to be abundant and promising sources of novel antibacterial, antiviral, anti-inflammatory, antiplasmodial and anticancer agent [16]. Among these, the diketopiperazines have various therapeutically important biological properties such as antitumor, antiviral, antifungal, and antihyperglycemic activities [17]. In our continuous search for bioactive secondary metabolites, we have isolated two natural diketopiperazine-type indole alkaloids, neoechinulins A (**1**) and B (**2**), from the marine fungus *Eurotium* sp. SF-5989.

In this study, we report the isolation and investigation of the structure-activity relationships of neoechinulin A (**1**) and neoechinulin B (**2**) by using assay to appraise anti-inflammatory effects in the LPS-induced inflammatory response in RAW 264.7 macrophages.

## 2. Results and Discussion

### 2.1. Identification of Neoechinulins A (**1**) and B (**2**)

**Figure 1:**
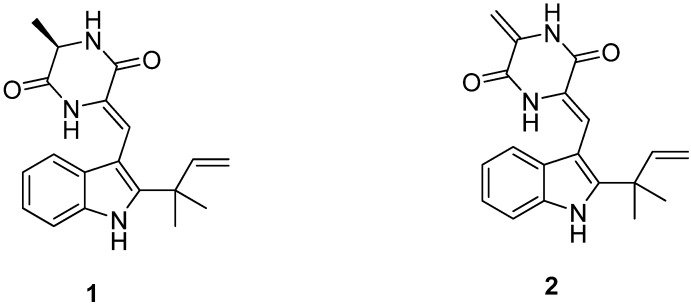
Chemical structures of neoechinulins A (**1**) and B (**2**).

To isolate and identify the anti-inflammatory metabolite(s) from the organic extract of the cultures of the marine fungus *Eurotium* sp. SF-5989, we performed bioassay- and ^1^H-NMR-guided fractionation and purification using C_18_-functionalized silica gel column chromatography and HPLC, which led to the isolation of two diketopiperazine type indole alkaloids, neoechinulins A (**1**) and B (**2**) (Figure 1). The structures of the isolated compounds were identified by analysis of NMR and MS data, coupled with comparison of their spectral data with those in the literature [18,19].

### 2.2. Effects of Neoechinulins A (**1**) and B (**2**) on Cell Viability and Pro-Inflammatory Mediators/Cytokines Production

**Figure 2:**
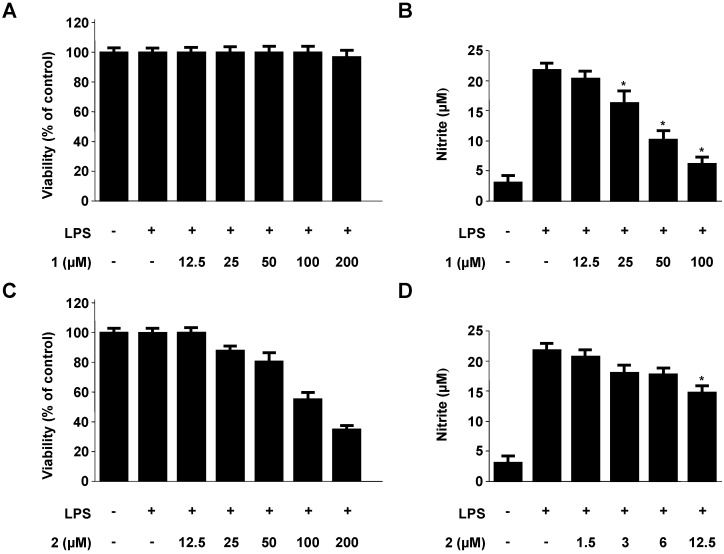
Effects of neoechinulins A (**1**) and B (**2**) on cell viability (A, C) and nitrite production (B, D). In the cell viability assay, RAW264.7 macrophages were incubated for 24 h with various concentrations (12.5 μM–200 μM) of neoechinulins A (**1**) and B (**2**). Cell viability was determined, as described in the materials and methods. In the nitrite production assay, RAW264.7 macrophages were pre-treated with indicated concentrations of neoechinulins A (**1**) and B (**2**) for 3 h, and then stimulated with LPS (1 μg/mL) for 18 h. The concentration of nitrite was determined as described in the Experimental. Data shown represent the mean values of three experiments ± SD. ^*^p < 0.05 as compared to the group treated with LPS alone.

Firstly, we evaluated the cytotoxicity and inhibitory effects of neoechinulins A (**1**) and B (**2**) on NO production in RAW264.7 macrophages. As shown in Figure 2A, the viability of the cells incubated with varying concentrations of **1** (12.5 µM–100 µM) was not affected significantly. In this concentration range, compound **1** suppressed NO production in a dose dependent manner (Figure 2B). Neoechinulin B (**2**) also inhibited NO production in a range from 1.5 µM to 12.5 µM, however, cell viability was significantly affected at concentrations exceeding 25 µM (Figure 2C and D). It is noteworthy that compound **2** has a much narrower therapeutic index than compound **1** in spite of their structural similarity. It has been suggested that the *α,β*-unsaturated carbonyl functionality as in neoechinulin B (**2**) is especially reactive and can interact with biological molecules, leading to a variety of adverse effects, such as general toxicity, allergenic reactions, and carcinogenicity [20]. Therefore, neoechinulin A (**1**) was proceeded to examine the anti-inflammatory properties.

There are several studies which indicate that inflammation is accompanied by increased NO production [21,22]. NO, synthesized by inducible nitric oxide synthase (iNOS), is a well-known pro-inflammatory mediator [23,24]. PGE_2_ is a major COX-2-derived product at inflammatory sites, which is induced during the response to various stimulants, and can trigger the development of inflammatory diseases [25,26]. Furthermore, pro-inflammatory cytokines, such as TNF-α and IL-1β, are also produced in response to inflammatory stimuli and can in turn contribute to inflammation [27]. Therefore, pro-inflammatory mediators and cytokines, such as NO, PGE_2_, TNF-α and IL-1β, are regarded as targets for inhibiting the inflammatory process.

**Figure 3:**
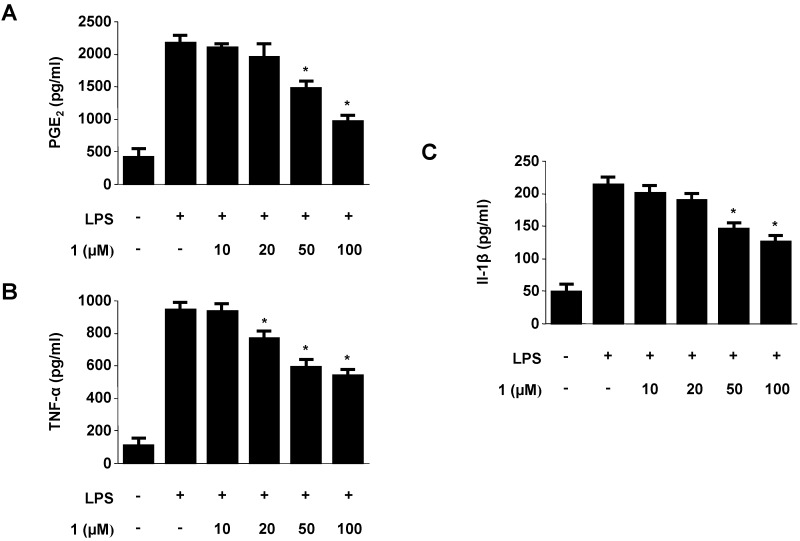
Effect of neoechinulin A (**1**) on the production of PGE_2_ (A) TNF-α (B), and IL-1β (C). RAW264.7 macrophages were pre-treated with the indicated concentrations of neoechinulin A for 3 h, and then stimulated with LPS (1 μg/mL) for 18 h. The concentrations of PGE_2_ (A) TNF-α (B), and IL-1β (C) were determined as described in the Experimental. Data shown represent the mean values of three experiments ± SD. ^* ^*p* < 0.05 as compared to the group treated with LPS alone.

To confirm the effect of neoechinulin A (**1**) on the production of pro-inflammatory mediators and cytokines, such as PGE_2_, TNF-α, and IL-1β, cells were stimulated with LPS (1 μg/mL) for 18 h in the presence or absence of non-cytotoxic concentrations of neoechinulin A (**1**). As shown in Figure 3, neoechinulin A (**1**) suppressed PGE_2_, TNF-α, and IL-1β production in a dose-dependent manner.

### 2.3. Effect of Neoechinulin A (**1**) on iNOS and COX-2 Expression

**Figure 4:**
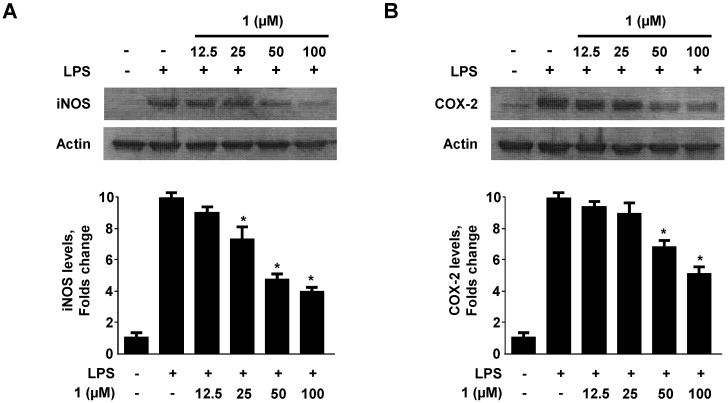
Effect of neoechinulin A (**1**) on the expression of iNOS (A) and COX-2 (B) protein. RAW264.7 macrophages were pre-treated with the indicated concentrations of neoechinulin A (**1**) for 3 h, and then treated with LPS (1 μg/mL) for 18 h. Representative blots from three independent experiments with similar results and densitometric evaluations are shown. Data shown represent the mean values of three experiments ± SD.^* ^*p* < 0.05 as compared to the group treated with LPS alone.

To confirm that the inhibition of NO and PGE_2 _production by neoechinulin A (**1**) is due to a decrease of iNOS and COX-2 protein expression, we examined the levels of iNOS and COX-2 protein expression after treating LPS-stimulated RAW264.7 macrophages with neoechinulin A (**1**). As shown in Figure 4, neoechinulin A (**1**) decreased iNOS and COX-2 protein expression in a dose-dependent manner.

### 2.4. Effect of Neoechinulin A (**1**) on NF-κB Activation

**Figure 5:**
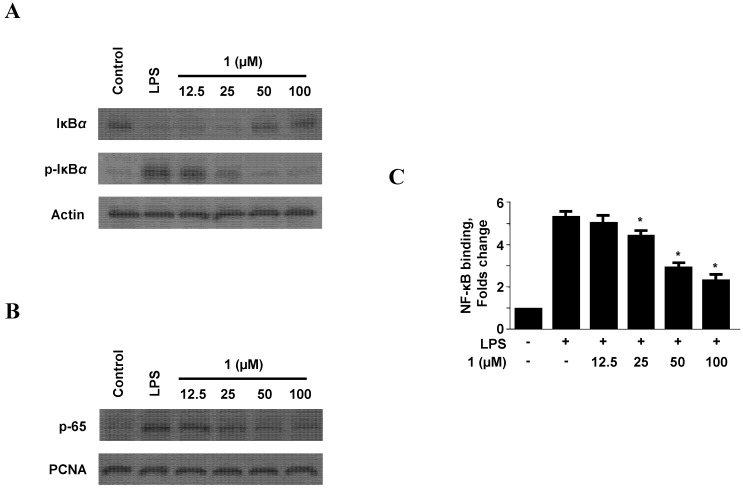
Effect of neoechinulin A (**1**) on IκB-α phosphorylation and degradation (A), NF-κB activation (B), and NF-κB DNA-binding activity (C). RAW264.7 macrophages were pre-treated with the indicated concentrations of neoechinulin A (**1**) for 3 h, and then stimulated with LPS (1 μg/mL) for 30 min. Western blot analysis was performed as described in the Experimental, and representative blots of three independent experiments are shown. A commercially available NF-κB ELISA kit (Active Motif) was used to test nuclear extracts and determine the degree of NF-κB binding. Data shown represent the mean values of three experiments ± SD. ^* ^*p* < 0.05 as compared to the group treated with LPS alone.

NF-κB, which regulates a variety of genes such as iNOS, COX-2, TNF-α, and IL-1β the transcription factor, is a crucial mediator of the inflammatory responses induced by pro-inflammatory cytokines or LPS [28]. NF-κB is a good target for treating inflammatory diseases because many anti-inflammatory compounds exert their effects by inhibiting the NF-κB signaling pathway [29,30]. Therefore, we examined the effect of neoechinulin A (**1**) on the phosphorylation and degradation of IκB-α, an inhibitor that associates with NF-κB in the cytoplasm. Neoechinulin A (**1**) inhibited the LPS-induced phosphorylation and degradation of IκB-α (Figure 5A). IκB-α was degraded after treatment of RAW264.7 macrophages with LPS for 30 min, and this degradation was markedly inhibited by pre-treatment with 12.5–100 µM of neoechinulin A (**1**) for 3 h. Moreover, neoechinulin A (**1**) dose-dependently reduced the levels of nuclear p65 protein increased by LPS treatment (Figure 5B). Neoechinulin A (**1**) also suppressed the DNA binding activity of NF-κB in nuclear extracts from RAW264.7 macrophages stimulated with LPS for 30 min. Figure 5C shows that the treatment of LPS markedly increased NF-κB binding activity, however, neoechinulin A (**1**) inhibited NF-κB binding activity in a dose-dependent manner. In addition, we observed this effect is not subject of temporal changes because pre-treatment of neoechinulin A (**1**) for 6 h and 12 h did not induce any inhibitory effects on the DNA binding activity of NF-κB in nuclear extracts (Supplementary Figure 2A).

### 2.5. Effect of Neoechinulin A (**1**) on the Phosphorylation of MAPKs

The MAPK pathway is known to be involved in the inflammatory process [31], and the inhibition of MAPK pathway is sufficient to block the induction of pro-inflammatory mediators by LPS [32]. Therefore, MAPKs, and p38 in particular, is regarded as an important target for the development of anti-inflammatory agents, as it has been implicated in the regulation of various inflammatory processes [33].

**Figure 6:**
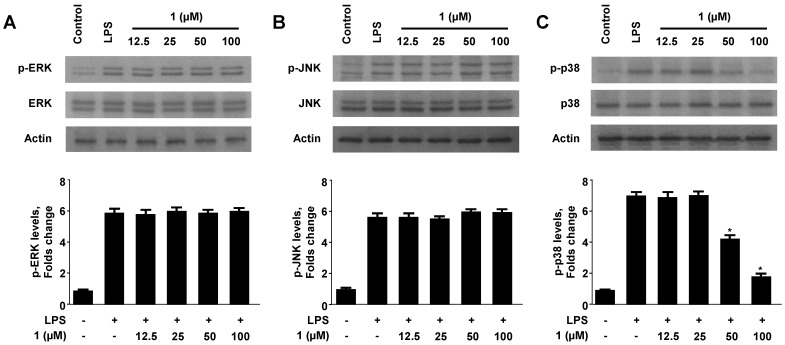
Effect of neoechinulin A (**1**) on ERK, JNK, and p38 MAPK phosphorylation and protein expression. RAW264.7 macrophages were pre-treated for 3 h with the indicated concentrations of neoechinulin A (**1**), and stimulated for 30 min with LPS (1 μg/mL). The levels of (A) phosphorylated-ERK (p-ERK), (B) phosphorylated-JNK (p-JNK), and (C) phosphorylated-p38 MAPK (p-p38 MAPK) were determined by performing Western blotting. Representative blots from three independent experiments with similar results and densitometric evaluations are shown. Data shown represent the mean values of three experiments ± SD. ^* ^*p* < 0.05 as compared to the group treated with LPS alone.

To investigate whether the suppression of inflammatory reactions by neoechinulin A (**1**) was mediated through the MAPK pathway, we assessed the effect of neoechinulin A (**1**) on the LPS-induced phosphorylation of ERK, JNK, and p38 in RAW264.7 macrophages. As shown in Figure 6, ERK, JNK, and p38 phosphorylation was increased after treatment with LPS for 30 min. However, pre-treatment with neoechinulin A (**1**) for 3 h, at 12.5 μM to 100 μM, significantly inhibited the LPS-induced p38 phosphorylation in a dose-dependent manner (Figure 6C), while ERK and JNK phosphorylation was not affected. On the other hand, the expression of ERK, JNK, and p38 were unaffected by LPS or neoechinulin A (**1**). These data suggested that neoechinulin A (**1**) regulated inflammatory reactions by inhibiting p38 MAPK signaling. In addition, we also observed this effect is not subject of temporal changes because pre-treatment of neoechinulin A (**1**) for 6 h and 12 h did not induce any inhibitory effects on the p38 phosphorylation (Supplementary Figure 2B).

## 3. Experimental

### 3.1. General

ESIMS data were obtained using a Q-TOF micro LC-MS/MS instrument (Waters, Manchester, U.K.) located at Korea University, Seoul, Korea. Optical rotations were recorded using a Jasco p-2000 digital polarimeter. NMR spectra (1D and 2D) were recorded in acetone-*d*_6_ with a JEOL JNM ECP-400 spectrometer (400 MHz for ^1^H and 100 MHz for ^13^C), and the chemical shifts were referenced relative to the residual solvent peaks (δ_H_/δ_C_ = 2.04/29.0). HSQC and HMBC experiments were optimized for ^1^*J*_CH_ = 140 Hz and ^n^*J*_CH_ = 8 Hz, respectively. The solvents for the extraction and flash column chromatography were reagent grade without further purification, while the solvents used for HPLC were analytical grade. Flash column chromatography was performed using YMC octadecyl-functionalized silica gel (C_18_). HPLC (YOUNGLIN-YL9100, Younglin, Anyang, Korea) separation was performed using a Shiseido Capcell Pak C_18_ column (20 × 150 mm, 5-mm particle size) with a flow rate of 5 mL/min.

Dulbecco’s modified Eagle’s medium (DMEM), fetal bovine serum (FBS), and other tissue culture reagents were purchased from Gibco BRL Co. (Grand Island, NY, USA). All chemicals were obtained from Sigma Chemical Co. (St. Louis, MO, USA). Antibodies to iNOS, COX-2, phosphor (p)-IκBα, IκBα, p65, PCNA and actin were obtained from Santa Cruz Biotechnology (Santa Cruz, CA, USA), and p-ERK, ERK, p-JNK, JNK, p-p38, p38 antibodies were obtained from Cell Signaling Technology (Cell Signaling, Danvers, MA, USA). Enzyme-linked immunosorbent assay (ELISA) kits for PGE_2_, TNF-α, and IL-1β were purchased from R & D Systems, Inc. (Minneapolis, MN, USA).

### 3.2. Specimen Collection and Identification of the Marine-Derived Fungus Eurotium sp. SF-5989

*Eurotium* sp. SF-5989 (deposited at the College of Medical and Life Sciences fungal strain repository, Silla University) was isolated from an unidentified soft coral that was manually collected using scuba equipment at a depth of 4.5–21 m at Terra Nova bay (74, 37' 39.895" S, 164, 14' 26.895" E), Antarctica in January 2012. The sample was stored in a sterile plastic bag and transported to the laboratory, where it was kept frozen until further processing. The sample was diluted 10 times with sterile seawater, and the ground sample was diluted 10-fold with sterile seawater. One mL of the diluted sample was processed utilizing the spread plate method in potato dextrose agar (PDA) medium containing 3% NaCl. The plate was incubated at 25 °C for 14 days. After purifying the isolates several times, the final pure cultures were selected and preserved at −70 °C. This fungus was identified based on the analysis of the ribosomal RNA (rRNA) sequence. A GenBank search with the 28S rRNA gene of SF-5989 (GenBank accession number KF573431) indicated *Eurotium rubrum* (AY004346), *Eurotium repens* (FR839678), *Aspergillus proliferans* (FR848827), *Eurotium chevalieri* (JN938915), *Eurotium amstelodami* (JN938912), *Eurotium herbariorum* (JN938918), and *Eurotium niveoglaucus* (HE578069) as the closest maches showing sequence identities of 100%, 99.48%, 99.48%, 99.48%, 99.48%, 99.48%, and 99.35%, respectively. Therefore, the marine-derived fungal strain SF-5989 was characterized as *Eurotium* sp.

### 3.3. Fermentation, Extraction and Isolation of Neoechinulins A (**1**) and B (**2**) from Eurotium sp. SF-5989

The fungal strain was cultured on fifty Petri-dishes (90 mm), each containing 20 mL of potato dextrose agar media [0.4% (w/v) potato starch, 2% (w/v) dextrose, 3% (w/v) NaCl, 1.5% (w/v) agar]. Plates were individually inoculated with 2 mL seed cultures of the fungal strain. Plated cultures were incubated at 25 °C for a period of 14 days. Extraction of the agar media with MEK (4 × 1000 mL) provided an organic phase, which was then concentrated in vacuo to yield 1.4 g of extract. The EtOAc extract was subjected to C_18_-functionalized silica gel flash column chromatography and eluted with a stepwise gradient of 20%, 40%, 60%, 80%, and 100% (*v/v*) of MeOH in H_2_O (500 mL each). The fraction (90 mg) eluted with 80% MeOH was further purified by using semi-preparative reversed phase HPLC with a gradient from 60% to 85% MeOH in H_2_O over 30 min to obtain neoechinulin A (**1**, 31.6 mg, t_R_ = 16.4 min) and neoechinulin B (**2**, 11.0 mg, t_R_ = 19.9 min).

*Neoechinulin A* (**1**). A white powder; the purity of isolated neoechinulin A (1) was greater than 98.0% as determined by HPLC analysis (data not shown); [α]_D_ −60° (c 0.2, MeOH); ^1^H-NMR data: δ 10.30 (1H, s, H-1), 7.39 (1H, d, *J* = 8.0 Hz, H-4), 7.18 (1H, dd, *J* = 7.8, 7.5 Hz, H-5), 7.16 (1H, dd, *J* = 7.5, 7.3 Hz, H-6), 7.29 (1H, d, *J* = 8.1Hz, H-7), 7.04 (1H, s, H-8), 7.91 (1H, br s, H-11), 4.27 (1H, qd, *J* = 7.0, 1.7 Hz, H-12), 6.66 (1H, s, H-14), 6.13 (1H, dd, *J* = 7.0, 10.6 Hz, H-16), 5.10(1H, d, *J* = 11.0 Hz, H_a_-17), 5.06 (1H, d, *J* = 4.0 Hz, H_b_-17), 1.55 (6H, s , H_3_-18/19), 1.51 (3H, d, *J* = 7.0 Hz, H_3_-20); ^13^C-NMR data: δ 144.8 (C-2), 104.4 (C-3), 127.3 (C-3a), 112.4 (C-4), 120.7 (C-5), 122.2 (C-6), 119.7 (C-7), 136.3 (C-7a), 110.6 (C-8), 126.7 (C-9), 160.3 (C-10), 52.2 (C-12), 166.9 (C-13), 40.1 (C-15), 146.0 (C-16), 112.3 (C-17), 27.9 (C-18), 27.9 (C-19), 20.7 (C-20); HRESIMS: *m/z* 322.1540 [M-H]^−^ (calcd for C_19_H_20_N_3_O_2_, 322.1556).

*Neoechinulin B* (**2**). Yellow brown solid; ^1^H-NMR data: δ 10.37 (1H, s, H-1), 7.36 (1H, d, *J* = 7.7 Hz, H-4), 7.05–7.14 (m, 2H, H-5 and H-6) 7.41 (1H, d, *J* = 7.7 Hz, H-7), 7.13 (1H, s, H-8), 9.78 (1H, br s, H-11), 8.13 (1H, s, H-14), 6.15 (1H, dd, *J* = 6.96 and 10.6 Hz, H-16), 5.12 (1H, d, *J* = 9.9 Hz, H_a_-17), 5.08 (1H, d, *J* = 3.3 Hz, H_b_-17), 1.56 (6H, s, H_3_-18/19), 5.02 (1H, s, H_a_-20), 5.36 (1H, s, H_b_-20); ^13^C-NMR data: δ 145.3 (C-2), 104.2 (C-3), 127.1 (C-3a), 112.5 (C-4), 120.9 (C-5), 122.3 (C-6), 119.7 (C-7), 136.3 (C-7a), 112.0 (C-8), 136.0 (C-9), 156.5 (C-10), 136.1 (C-12), 157.7 (C-13), 40.1 (C-15), 145.8 (C-16), 112.4 (C-17), 27.9 (C-18), 27.9 (C-9), 100.0 (C-20); HRESIMS: *m/z* 320.1411 [M-H]^−^ (calcd for C_19_H_18_N_3_O_2_, 320.1399).

### 3.4. Cell Culture and Viability Assay

RAW264.7 macrophages were maintained at a density of 5 × 10^5^ cells/mL in DMEM medium supplemented with 10% heat-inactivated FBS, penicillin G (100 units/mL), streptomycin (100 mg/mL), and l-glutamine (2 mM), and were incubated at 37 °C in a humidified atmosphere containing 5% CO_2_. The effect of the various experimental treatments on cell viability were evaluated by determining mitochondrial reductase function with an assay based on the reduction of the tetrazolium salt 3-[4,5-dimethylthiazol-2-yl]-2,5-diphenyltetrazolium bromide (MTT) into fomazan crystals [34]. The formation of formazan is proportional to the number of functional mitochondria in the living cells. For the determination of cell viability, 50 µL of MTT (2.5 mg/mL) was added to cell suspension (1 × 10^5^ cells/mL in each well of the 96-well plates) at a final concentration of 0.5 mg/mL, and the mixture was further incubated for 3–4 h at 37 °C. The formazan formed was dissolved in acidic 2-propanol, and the optical density was measured at 590 nm. The optical density of the formazan formed in the control (untreated) cells was considered as 100% viability.

### 3.5. Determination of Nitrite Production and PGE_2_, TNF-α, and IL-1β Assays

The production of nitrite, a stable end product of NO oxidation, was used as a measure of iNOS activity. The nitrite present in the conditioned medium was determined using a method based on the Griess reaction [35]. The concentrations of PGE_2_, TNF-α, and IL-1β in the culture medium were determined using ELISA kits (R&D Systems) according to the manufacturer’s instructions.

### 3.6. Preparation of Cytosolic and Nuclear Fractions

RAW264.7 macrophages were homogenized in PER-Mammalian Protein Extraction Buffer (1: 20, w: v) (Pierce Biotechnology, Rockford, IL, USA) containing freshly added protease inhibitor cocktail I (EMD Biosciences, San Diego, CA, USA) and 1 mM PMSF. The cytosolic fraction of the cells was prepared by centrifugation at 15,000 × *g* for 10 min at 4 °C. Nuclear and cytoplasmic extracts of cells were prepared using NE-PER nuclear and cytoplasmic extraction reagents (Pierce Biotechnology), respectively.

### 3.7. Western Blot Analysis

RAW264.7 macrophages were harvested and pelleted by centrifugation at 200 × *g* for 3 min. Then, the cells were washed with PBS and lysed in 20 mM Tris-HCl buffer (pH 7.4) containing a protease inhibitor mixture (0.1 mM phenylmethanesulfonyl fluoride, 5 mg/mL aprotinin, 5 mg/mL pepstatin A, and 1 mg/mL chymostatin). Protein concentration was determined using a Lowry protein assay kit (P5626; Sigma). Thirty μg of protein from each sample was resolved by 12% sodium dodecyl sulfate-polyacrylamide gel electrophoresis (SDS-PAGE), and then electrophoretically transferred onto a Hybond enhanced chemiluminescence (ECL) nitrocellulose membrane (Bio-Rad, Hercules, CA, USA). The membrane was blocked with 5% skimmed milk and sequentially incubated with the primary antibody (Santa Cruz Biotechnology and Cell Signaling Technology) and a horseradish peroxidase-conjugated secondary antibody followed by ECL detection (Amersham Pharmacia Biotech, Piscataway, NJ, USA).

### 3.8. DNA Binding Activity of NF-κB

The DNA-binding activity of NF-κB in nuclear extracts was measured using the TransAM kit (Active Motif, Carlsbad, CA, USA) according to the manufacturer’s instructions. Briefly, 30 μL of complete binding buffer (DTT, herring sperm DNA, and binding buffer AM3) was added to each well. The samples were nuclear extracts from RAW264.7 macrophages stimulated for 30 min with LPS and treated with different-concentrations of neoechinulin A (**1**). Then, 20 μL of the samples in complete lysis buffer were added to each well (20 μg of nuclear extract diluted in complete lysis buffer). The plates were incubated for 1 h at room temperature with mild agitation (100 rpm on a rocking platform). After washing each well with wash buffer, 100 μL of diluted NF-κB antibody (1:1000 dilution in 1× antibody binding buffer) was added to each well, and then the plates were incubated further for 1 h as before. After washing each well with wash buffer, 100 μL of diluted HRP-conjugated antibody (1:1000 dilution in 1× antibody binding buffer) was added to each well, followed by 1 h incubation as before. One hundred μL of developing solution was added to each well for 5 min, followed by the addition of stop solution. Finally, the absorbance of each sample at 450 nm was determined with a spectrophotometer within 5 min.

### 3.9. Statistical Analysis

Data was expressed as the mean ± SD of at least three independent experiments. To compare three or more groups, one-way analysis of variance followed by the Newman-Keuls post hoc test was used. Statistical analysis was performed with GraphPad Prism software, version 3.03 (GraphPad Software Inc., San Diego, CA, USA).

## 4. Conclusions

Bioassay-guided chemical investigation of the marine-derived fungus *Eurotium* sp. SF-5989 afforded two diketopiperazine-type indole alkaloids neoechinulins A (**1**) and B (**2**) as anti-inflammatory compounds. Prior investigations had resulted in the isolation of anthraquinone derivatives [36], dioxopiperazine alkaloids [37], and benzaldehyde derivatives [38] from the marine endophytic fungus *Eurotium rubrum*. Neoechinulins A and B were first isolated from *Aspergillus amstelodami* [39] and described as secondary metabolites from *Eurotium* species [40]. In the previous studies, neoechinulin A has been demonstrated to have anti-proliferative activity toward various tumor cells [41], protective effects in neuronal cells [42,43], anti-oxidant activity [44], radical scavenging activity, and ultraviolet-A protecting activity [45], pro-inflammatory activity in the lungs [46], and anti-neuroinflammatory activity in BV2 cells [47]. In addition, the study of Rand *et al.* showed differential effects of neoechinulins A and B on the pro-inflammatory gene transcription and expression in primary mouse alveolar macrophages [48]. However, to our best knowledge, there are no reports to date on the mechanism underlying the anti-inflammatory effects of neoechinulins in RAW264.7 macrophage cells.

Bioassay-guided chemical investigation of the marine-derived fungus *Eurotium* sp. SF-5989 afforded two diketopiperazine-type indole alkaloids neoechinulins A (**1**) and B (**2**) as anti-inflammatory compounds. Prior investigations had resulted in the isolation of anthraquinone derivatives [36], dioxopiperazine alkaloids [37], and benzaldehyde derivatives [38] from the marine endophytic fungus *Eurotium rubrum*. Neoechinulins A and B were first isolated from *Aspergillus amstelodami* [39] and described as secondary metabolites from *Eurotium* species [40]. In the previous studies, neoechinulin A has been demonstrated to have anti-proliferative activity toward various tumor cells [41], protective effects in neuronal cells [42,43], anti-oxidant activity [44], radical scavenging activity, and ultraviolet-A protecting activity [45], pro-inflammatory activity in the lungs [46], and anti-neuroinflammatory activity in BV2 cells [47]. In addition, the study of Rand *et al.* showed differential effects of neoechinulins A and B on the pro-inflammatory gene transcription and expression in primary mouse alveolar macrophages [48]. However, to our best knowledge, there are no reports to date on the mechanism underlying the anti-inflammatory effects of neoechinulins in RAW264.7 macrophage cells.

In this study, we evaluated the anti-inflammatory effects and mechanism of action of neoechinulin A (**1**). It was shown that neoechinulin A (**1**) can suppress the production of pro-inflammatory mediators and cytokines including NO, PGE_2_, TNF-α, and IL-1β. Further, it reduced the expression of iNOS and COX-2 in LPS-stimulated RAW264.7 macrophages by inhibiting the NF-κB and p38 MAPK signaling pathways. Because a variety of signaling pathways are involved in the mediation of inflammatory responses, further studies are necessary to investigate whether other signaling pathways are involved in modulating the inflammatory response to neoechinulin A.

## Supplementary Materials

Supplementary materials can be accessed at: http://www.mdpi.com/1420-3049/18/11/13245/s1.
