# Supplementary material for: Anti-Inflammatory Effect of Neoechinulin A from the Marine Fungus Eurotium sp. SF-5989 through the Suppression of NF-кB and p38 MAPK Pathways in Lipopolysaccharide-Stimulated RAW264.7 Macrophages
Source: Molecules. 2013 Oct 25;18(11):13245–59. doi: 10.3390/molecules181113245 (PMC6270177; doi:10.3390/molecules181113245)

Supplementary Materials

Figure S1. The effects of neoechinulin A (**1**) on the cell proliferation. The cell viability (A) and the morphology (x200) (B) of RAW264.7 macrophages. RAW264.7 macrophages were incubated for 48 h with various concentrations of neoechinulin A (12.5–200 μM). Cell viability was determined as described in the Experimental. The data represent the mean values of three experiments ± S.D.

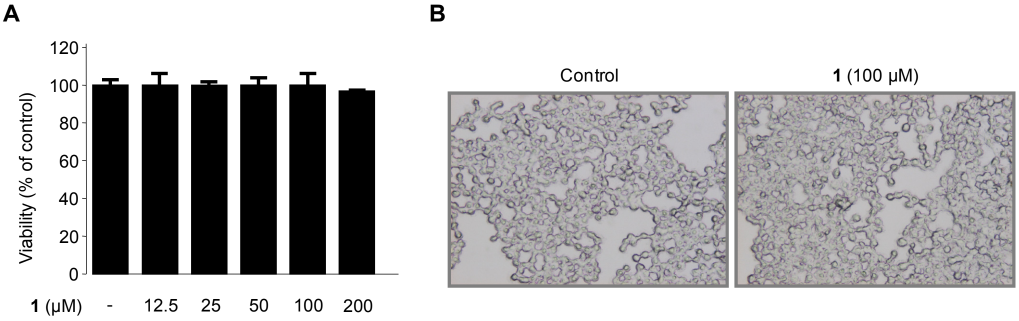

Figure S2. Effect of neoechinulin A (**1**) on NF-κB DNA-binding activity (A) and p38 MAPK phosphorylation (B). RAW264.7 macrophages were pre-treated with the indicated concentrations of neoechinulin A (1) for 3 h, 6 h, and 12 h, and then stimulated with LPS (1 μg/mL) for 30 min. A commercially available NF-κB ELISA kit (Active Motif) was used to test nuclear extracts and determine the degree of NF-κB binding. The phosphorylated-p38 MAPK (p-p38 MAPK) were determined by performing Western blotting. The densitometric evaluations of proteins were obtained from three different experiments. Data shown represent the mean values of three experiments ± S.D. $^{*}p < 0.05$ as compared to the group treated with LPS alone.

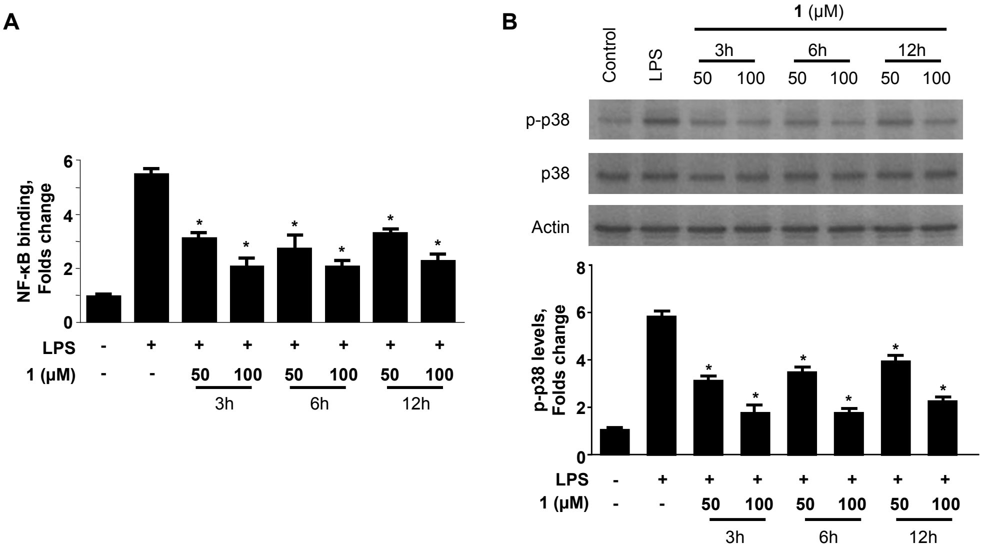

Supplement: Supplementary file 1 [file molecules-18-13245-s001.pdf]
